# Rapid Classification of Serum from Patients with Paracoccidioidomycosis Using Infrared Spectroscopy, Univariate Statistics, and Linear Discriminant Analysis (LDA)

**DOI:** 10.3390/jof10020147

**Published:** 2024-02-12

**Authors:** Alessandra Koehler, Maria Lúcia Scroferneker, Nikolas Mateus Pereira de Souza, Paulo Cezar de Moraes, Beatriz Aparecida Soares Pereira, Ricardo de Souza Cavalcante, Rinaldo Pôncio Mendes, Valeriano Antonio Corbellini

**Affiliations:** 1 Postgraduate Program of Medicine: Medical Sciences, Universidade Federal do Rio Grande do Sul-UFRGS, Porto Alegre 90035-003, Brazil; aleskoehler@gmail.com (A.K.); scrofern@ufrgs.br (M.L.S.); mpchansen@gmail.com (P.C.d.M.); 2 Department of Microbiology, Immunology and Parasitology, ICBS, Universidade Federal do Rio Grande do Sul-UFRGS, Porto Alegre 90050-170, Brazil; 3 Department of Life Sciences, Universidade de Santa Cruz do Sul-UNISC, Santa Cruz do Sul 96815-900, Brazil; nikolas1@mx2.unisc.br; 4 Tropical Diseases Area, School of Medicine, Universidade Estadual Paulista-UNESP, Botucatu 18618-687, Brazilmip.ricardo@gmail.com (R.d.S.C.); tietemendes@terra.com.br (R.P.M.); 5 Department of Sciences, Humanities and Education, Postgraduate Program in Health Promotion, Postgraduate Program in Environmental Technology, Universidade de Santa Cruz do Sul-UNISC, Santa Cruz do Sul 96815-900, Brazil

**Keywords:** paracoccidioidomycosis, Fourier transform infrared spectroscopy, photodiagnosis, ROC curve, linear discriminant analysis, systemic mycosis

## Abstract

Paracoccidioidomycosis (PCM) is a systemic mycosis that is diagnosed by visualizing the fungus in clinical samples or by other methods, like serological techniques. However, all PCM diagnostic methods have limitations. The aim of this study was to develop a diagnostic tool for PCM based on Fourier transform infrared (FTIR) spectroscopy. A total of 224 serum samples were included: 132 from PCM patients and 92 constituting the control group (50 from healthy blood donors and 42 from patients with other systemic mycoses). Samples were analyzed by attenuated total reflection (ATR) and a *t*-test was performed to find differences in the spectra of the two groups. The wavenumbers that had *p* < 0.05 had their diagnostic potential evaluated using receiver operating characteristic (ROC) curves. The spectral region with the lowest *p* value was used for variable selection through principal component analysis (PCA). The selected variables were used in a linear discriminant analysis (LDA). In univariate analysis, the ROC curves with the best performance were obtained in the region 1551–1095 cm^−1^. The wavenumber that had the highest AUC value was 1264 cm^−1^, achieving a sensitivity of 97.73%, specificity of 76.01%, and accuracy of 94.22%. The total separation of groups was obtained in the PCA performed with a spectral range of 1551–1095 cm^−1^. LDA performed with the eight wavenumbers with the greatest weight from the group discrimination in the PCA obtained 100% accuracy. The methodology proposed here is simple, fast, and highly accurate, proving its potential to be applied in the diagnosis of PCM. The proposed method is more accurate than the currently known diagnostic methods, which is particularly relevant for a neglected tropical mycosis such as paracoccidioidomycosis.

## 1. Introduction

Paracoccidioidomycosis (PCM) is a systemic granulomatous mycosis caused by thermodimorphic fungi of the genus *Paracoccidioides*. PCM is endemic in Latin America, from Mexico to Argentina. Approximately 80% of cases occur in Brazil, which has areas of high endemicity in the Southeast, South, and Center-West regions, with progression in the North region [1,2]. For many years, it was believed that the genus *Paracoccidioides* was composed of just one species, *P. brasiliensis*. However, with the advent of molecular techniques, it became clear that the genetic diversity of the genus was much greater than initially speculated. Therefore, it is currently thought that there are at least five species of the genus *Paracoccidioides* that can cause PCM: *P. brasiliensis*, *P. lutzii*, *P. restrepiensis*, *P. venezuelensis*, and *P. americana* [3,4]. *P. brasiliensis* is the most widely distributed species on the South American continent, followed by *P. lutzii* and *P. americana*. *P. restrepiensis* and *P. venezuelensis* have a more restricted distribution, mainly in Colombia and Venezuela, respectively, with some scattered cases in Argentina, Brazil, Peru, and Uruguay [5].

These fungi grow saprophytically as mycelium in the soil; however, due to the difficulty of their isolation from nature, their habitat is little known. The infection is acquired by inhaling fungal propagules present in the bioaerosol, which pass through the respiratory tract and reach the lungs, one of the main organs affected in PCM. If the immune response is insufficient, the fungus proliferates and spreads to other organs in the body via the lymphatic system and the hematogenous route. Other areas of the body commonly affected are the mucous membranes of the upper aerodigestive tract, the skin, and the adrenal glands [1,2].

The diagnosis and adequate treatment of PCM are essential, as it is highly related to several sequelae. It is estimated that at least 60% of patients with the chronic form of PCM will develop pulmonary fibrosis, the main sequela related to PCM [6]. The “gold standard” for PCM diagnosis is the visualization of the fungus in clinical samples, especially through direct examination with microscopy [7]. Other possibilities are through biopsy and histopathology and/or the isolation and cultivation of the fungus from clinical samples. However, these methods of conventional microbiological diagnosis from clinical samples may present low sensitivity and be time-consuming, in addition to the frequent difficulty in obtaining samples [8]. Another possibility is serological diagnostics, performed mainly with the double immunodiffusion (DID) technique. However, the lack of standardization of the technique and of the preparation of the antigens leads to discordant results. Furthermore, it is known that different species of the genus *Paracoccidioides* have different antigenic profiles; however, most tests are performed with *P. brasiliensis* antigens, which can lead to a considerable number of false negative results [9,10]. Last but not least, there is the possibility of a cross-reaction with other systemic mycoses such as histoplasmosis, aspergillosis, and cryptococcosis, with false positive results for PCM [11]. This risk can be minimized, but not excluded, with the standardization of the antigens used in tests [1].

Therefore, there is a need to develop new diagnostic methods, with an emphasis on some technologies, such as Matrix-Assisted Laser Desorption Ionization Time Of Flight Mass Spectrometry (MALDI-TOF) and Fourier transform infrared spectroscopy (FTIR). MALDI-TOF has a wide possibility of use in infectious diseases, and is used for identifying different species of bacteria and fungi [12]. For PCM, MALDI-TOF has already been successfully used to differentiate between the species *P. brasiliensis* and *P. lutzii* [13]. In addition to MALDI-TOF, FTIR has gained prominence in medicine for the diagnosis of several diseases. This technique provides qualitative and quantitative information on the compounds present in a sample, through the interaction of infrared radiation with the chemical bonds present in these compounds. In this way, a spectrum is generated that contains the fingerprint of the sample [14]. To validate FTIR data as a diagnostic tool, complex tools based on multivariate statistics (chemometrics) are mostly used, with the use of supervised classification methods [15]. FTIR has already been used for PCM diagnosis, using the machine learning algorithms discriminant analysis (DA), k-nearest neighbor (KNN), support vector machine (SVM) [16], and orthogonal partial least squares discriminant analysis (OPLS-DA) [17].

Here, we are proposing the use of FTIR spectroscopy, univariate statistics, and linear discriminant analysis (LDA) to differentiate the serum of patients with PCM from that of healthy individuals and patients with other systemic mycoses, in the same model. The main advantage here in relation to other studies that have already used FTIR to diagnose PCM is the careful selection of variables for multivariate modelling, developing a more robust model and showing its potential as a rapid diagnostic tool for PCM.

## 2. Materials and Methods

### 2.1. Serum Samples

A total of 224 retrospective serum samples from a repository at the School of Medicine of Botucatu, Universidade Estadual de São Paulo (UNESP), were analyzed. Of these, 132 samples were from patients with paracoccidioidomycosis treated at the Infectious Diseases Service of the School of Medicine of Botucatu (UNESP), from the Botucatu region, São Paulo, Brazil. The cases were confirmed and diagnosed after clinical suspicion, followed by visualization of the fungus of the genus *Paracoccidioides* in clinical samples, by direct mycological examination and/or culture and/or histopathology and/or cytopathology by cyto-inclusion [1]. These 132 samples constituted the group called PCM.

The other 92 serum samples consisted of the control group: 50 from healthy blood donors without suspected PCM at the Botucatu Blood Center, from the same region of origin as the PCM patients; 24 from patients with aspergillosis; 10 from patients with cryptococcosis; and 8 from patients with histoplasmosis. These other systemic mycoses can be confused with PCM in diagnostic tests that use serum, which justifies the importance of their inclusion in this study in the control group.

### 2.2. FTIR/ATR Analysis

Serum samples were analyzed in triplicate by attenuated total reflection (ATR) on a Spectrum 400 FT-IR/FT-NIR (Perkin Elmer, Waltham, USA) spectrometer, coupled to standard a Universal ATR Sampling Accessory (UATR, Perkin-Elmer Inc.; Registration number L1250050). Triplicates of 1 µL of each sample were deposited on the crystal of the instrument and dehydrated in an airstream (60–65 °C) for one minute. The acquisition range was from 4000 to 650 cm^−1^ using the spectral resolution of 4 cm^−1^ and 8 scans. Between the spectral acquisitions of each of the serum samples and their triplicates, a blank acquisition was performed under the same conditions.

### 2.3. Statistical Analysis

For univariate analyses, the average spectrum of each serum sample was obtained after vector normalization of the replicates in a Microsoft Excel spreadsheet (2019). The analyses were conducted in the software OriginPro 2018 (OriginLab, Northampton, MA, USA).

Initially, a *t*-test with a significance level of 0.05 was performed between the PCM group and the control group, to determine which wavenumbers differentiate the serum of patients with PCM from the other serum samples [18]. To verify its diagnostic potential, receiver operating characteristic (ROC) curves were generated for all wavenumbers individually, in the segments of the spectra that presented *p* < 0.05 in the *t*-test. The ROC curves with the best performance were chosen by analyzing the values of the area under the curve (AUC). The optimal cut points of the chosen ROC curve were determined according to the Youden index (J) [19], and the sensitivity and specificity parameters were determined considering these cut points.

For multivariate analyses, the average spectrum of each serum sample was obtained after the application of the 1st derivative (5 points), followed by vector normalization [15]. The analyses were conducted in the software The Unscrambler^®^ X 10.4 (Camo Analytics, Bedford, MA, USA).

Initially, a principal component analysis (PCA) with mean-centering was performed to reduce data dimensionality and select variables. For PCA, instead of using the full spectrum, a spectral range of 1551–1095 cm^−1^ was used, which was selected in the univariate analysis because it presented the highest AUC values in the ROC curve analysis. Therefore, PCA was used as a second step for variable selection. The wavenumbers that best discriminated the groups (PCM and control) in the PCA loadings were selected for the development of a classification model based on linear discriminant analysis (LDA). For LDA, the total dataset (*n* = 224) was systematically divided into a 1:1 ratio into a training set and prediction set, resulting in 112 serum samples in each set. The performance of the LDA model for diagnosing PCM was evaluated by parameters of sensitivity, specificity, and accuracy, obtained through the classification of the samples from the prediction set.

## 3. Results

**Figure 1:**
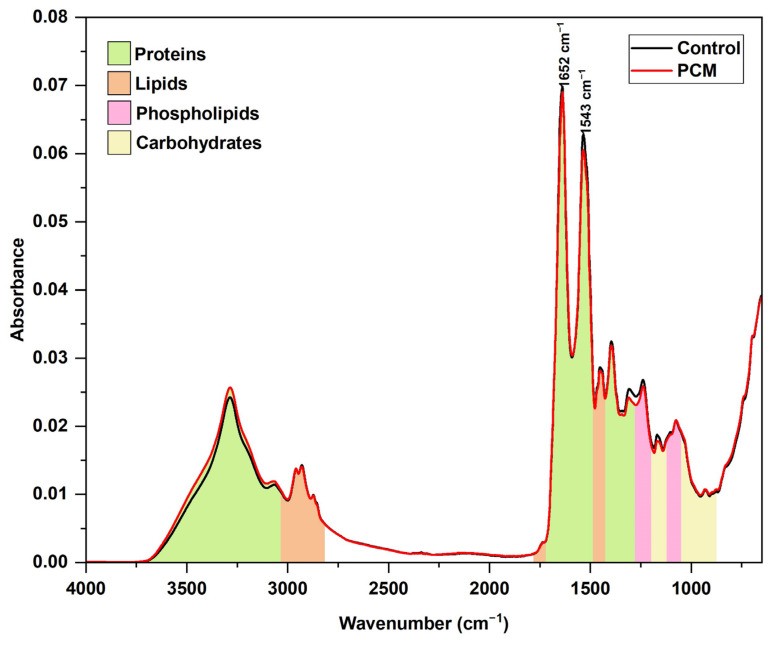
Average Fourier transform infrared (FTIR)/attenuated total reflection (ATR) spectra of serum from the 92 samples of the control group and from the 132 samples of the paracoccidioidomycosis (PCM) group. Averaging from each group was obtained by calculating the arithmetic mean of absorption for each frequency of the three vector-normalized spectra from each sample, followed by the arithmetic mean of all the samples from the group. The colors represent broad examples of blood serum constituents, based on Gray et al. (2018) [21]; they are not necessarily the only components that show absorption in that region of the spectrum.

The average spectra of the serum samples from the patients with PCM and the control group are shown in Figure 1. The main regions of the spectra are related to proteins and lipids, and the main bands are amide I and amide II, at approximately 1652 cm^−1^ and 1543 cm^−1^, respectively [20].

**Figure 2:**
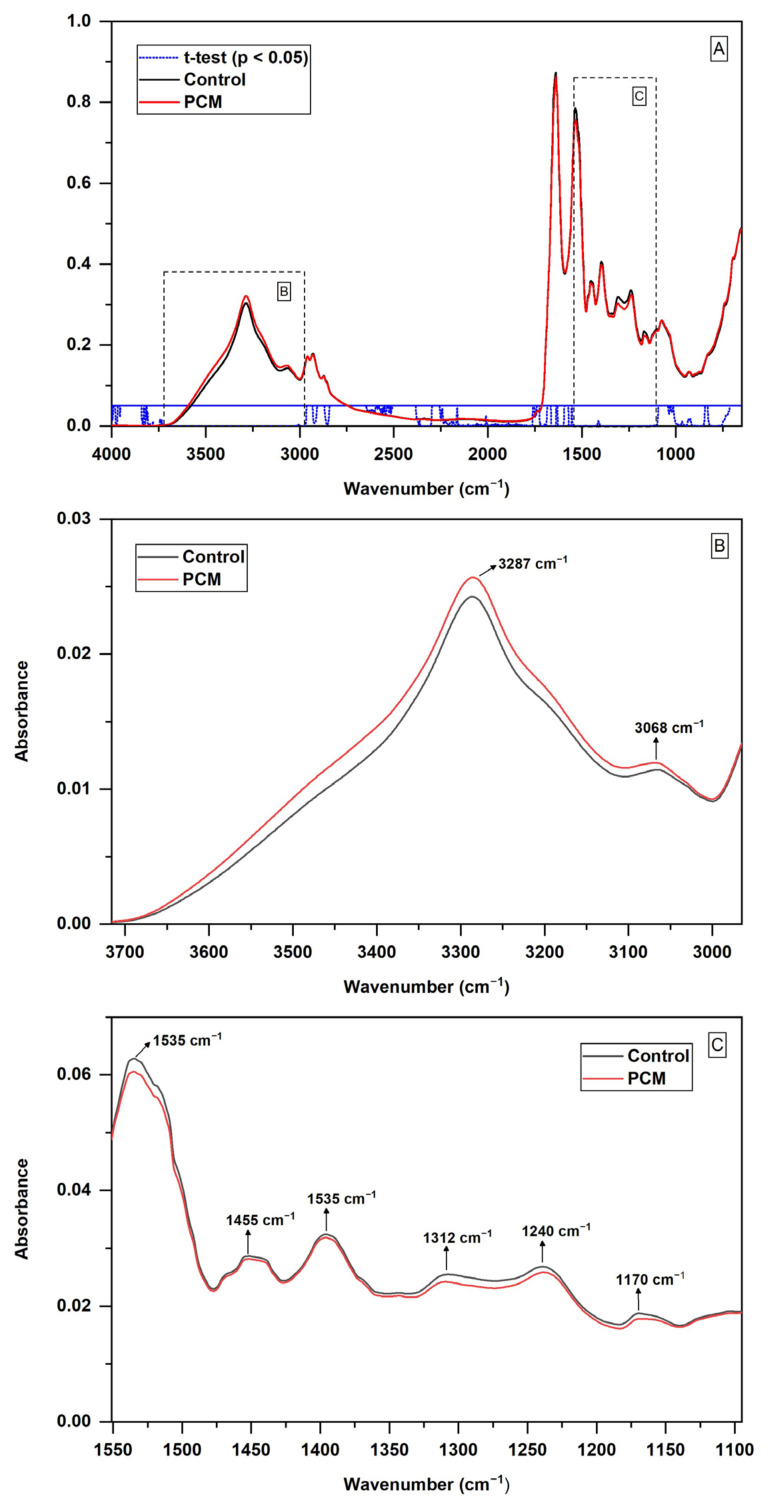
*t*-test performed between the averages of the spectra of the 92 serum samples of the control group and the 132 serum samples of the paracoccidioidomycosis (PCM) group, showing the main differences between the groups. Averaging from each group was obtained by calculating the arithmetic mean of absorption for each frequency of the three vector-normalized spectra from each sample, followed by the arithmetic mean of all the samples from the group. (**A**) Spectrum regions that had *p* < 0.05 in the *t*-test; (**B**) 3700–2965 cm^−1^ region enlarged, showing the differences between the two groups and the main bands found; (**C**) 1551–1095 cm^−1^ region enlarged, showing the differences between the two groups and the main bands found.

Several wavenumbers of the spectra had statistically significant differences (*p* < 0.05) in terms of their absorbance in the two groups analyzed (Figure 2A). The main regions, with the lowest *p* values, were 3700–2965 cm^−1^, mainly related to proteins, and 1551–1095 cm^−1^, mainly related to proteins, lipids, and phospholipids (Figure 2B and Figure 2C, respectively).

The diagnostic potential of the wavenumbers with *p* < 0.05 was evaluated using the ROC curve. This curve is a graphical plot of the sensitivity versus the (1-)specificity, determining several possible cut points for the test. The AUC is equivalent to test accuracy; the closer to 1, the greater the accuracy. From the ROC curve, it is possible to determine a cut point that maximizes the accuracy of the diagnostic test [22]. The ROC curves with the best performance were obtained in some of the wavenumbers in the region from 1551 to 1095 cm^−1^. All other spectral regions, even with *p* < 0.05 in the *t*-test (Figure 2A), had low AUC values, indicating that they do not have a good diagnostic potential for PCM (Table 1).

**Figure 3:**
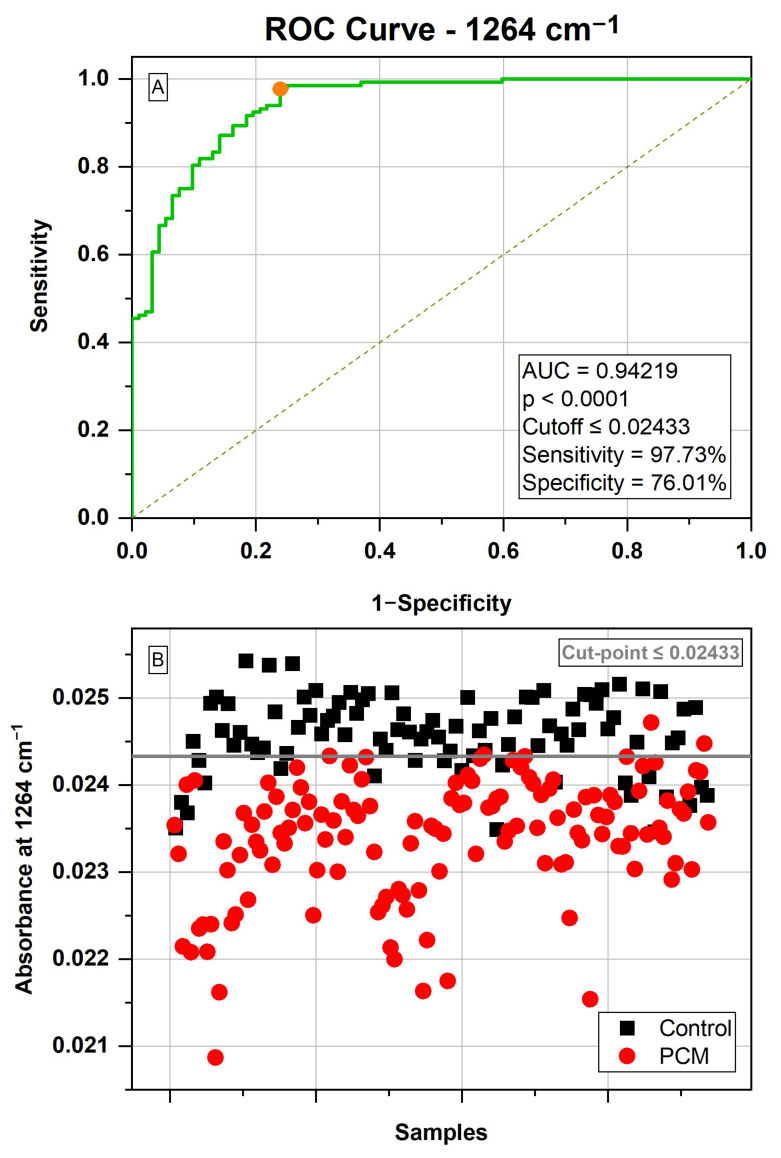
Potential of the wavenumber 1264 cm^−1^ to differentiate between the spectra of the serum samples from the control group (92 samples) and the serum samples from the paracoccidioidomycosis (PCM) group (132 samples). (**A**) Receiver operating characteristic (ROC) curve developed considering the wavenumber 1264 cm^−1^, with its respective parameters, including the area under the curve (AUC); the dot represents the selected cut point, defined based on the Youden index (J); (**B**) classification of samples from the control group (n = 92) and from the paracoccidioidomycosis (PCM) group (n = 132), considering a cut point ≤ 0.02433.

In the region of 1551–1095 cm^−1^, the wavenumber that had the highest AUC value was 1264 cm^−1^, with an AUC of 0.94219 and *p* < 0.0001. The optimal cut point was defined at ≤0.02433, resulting in a sensitivity of 97.73%, specificity of 76.01%, and accuracy of 94.22% (Figure 3).

**Figure 4:**
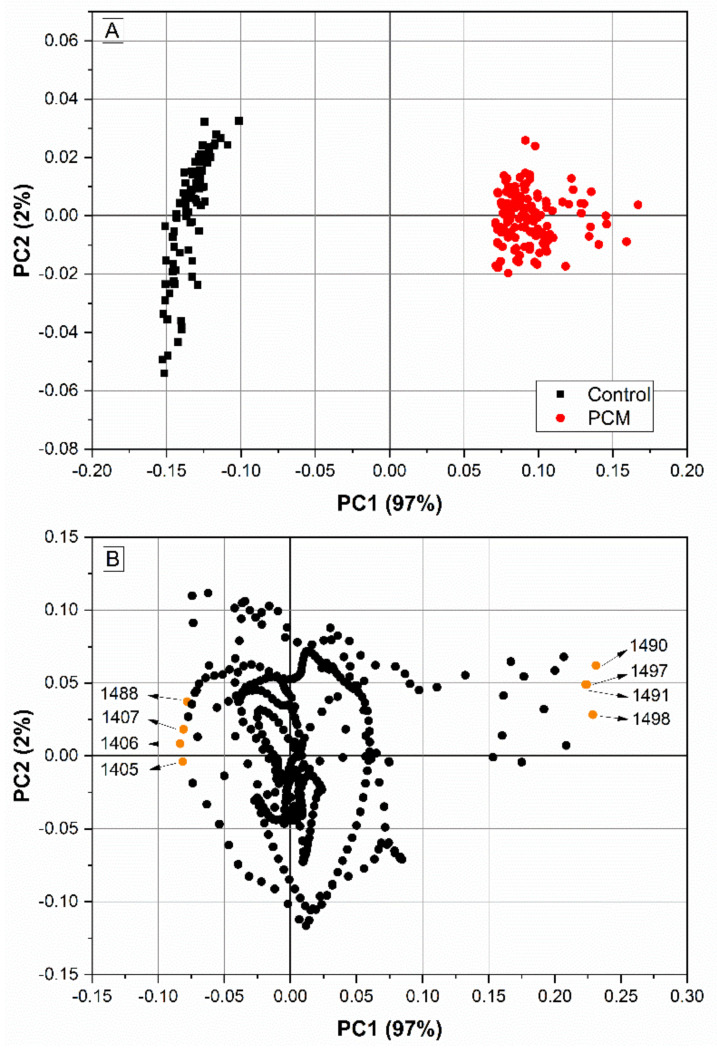
Principal component analysis (PCA) of the region 1551–1095 cm^−1^, performed with the spectra of the 92 serum samples of the control group and the 132 serum samples of the paracoccidioidomycosis (PCM) group. The control group is composed of serum from 50 healthy blood donors, 24 patients with aspergillosis, 10 patients with cryptococcosis, and 8 patients with histoplasmosis. This region was chosen because it was the interval with the largest area under the curve (AUC) to discriminate between PCM and the control group. (**A**) Scores (PC1 × PC2) with 99% explained variance. (**B**) Loadings (PC1 × PC2) indicating the variables with the greatest weight in group discrimination. The four wavenumbers (cm^−1^) most associated with the control and the four most associated with PCM are highlighted.

In the multivariate analysis, a total separation of the groups was obtained in the PCA performed with a spectral range of 1551–1095 cm^−1^ (Figure 4A). PC1 and PC2 represented 99% of the variance of the original data, with PC1 alone representing 97% of the variance, due to the high correlation of the original variables in the spectral range used. PC1 was responsible for separating the groups, and the variables with the greatest weight in group discrimination were 1490, 1491, 1497, and 1498 cm^−1^ for PCM and 1405, 1406, 1407, and 1488 cm^−1^ for the control group (Figure 4B).

**Figure 5:**
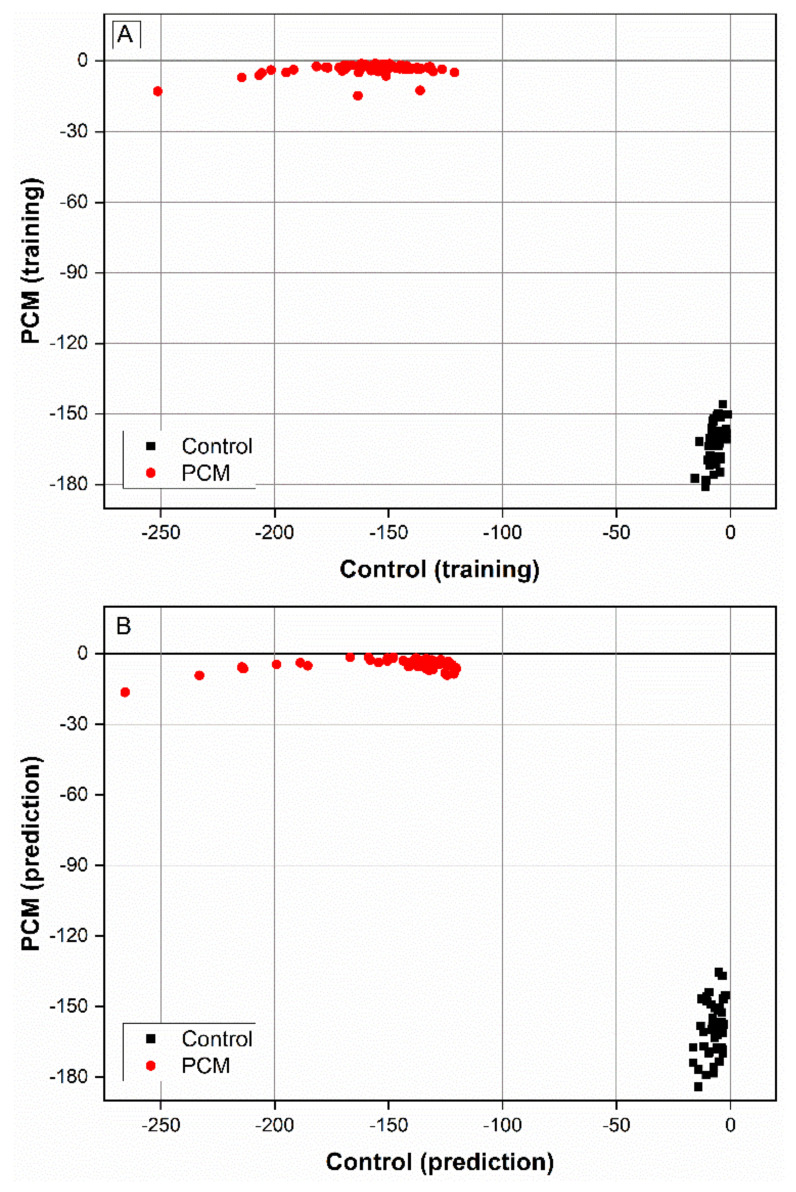
Linear discriminant analysis (LDA) used to discriminate between the spectra of the 92 serum samples of the control group and the 132 serum samples of the paracoccidioidomycosis (PCM) group. The model was constructed using the eight wavenumbers with the highest loadings in the principal component analysis (PCA) (1405, 1406, 1407, 1488, 1490, 1491, 1497, and 1498 cm^−1^) and obtained 100% accuracy both in the training set (**A**) and in the prediction set (**B**).

Linear discriminant analysis (LDA), performed with the eight wavenumbers with the greatest weight from the group discrimination in the PCA, obtained 100% sensitivity, specificity, and accuracy, both in the training set and in the prediction set (Figure 5A,B).

## 4. Discussion

The sensitivity and specificity parameters of routine PCM diagnostic methods vary significantly, depending mainly on the clinical sample used. In addition, these parameters are little discussed in the literature. An evaluation of the routine diagnostic methods in a university hospital over 34 years showed that direct mycological examination has a sensitivity ranging from 63% (from sputum) to 82.6% (from skin and mucous membrane samples). Histopathology reached a sensitivity of 96.7%; however, for none of the methods was the specificity discussed. Serological methods had better performances, with 90% sensitivity and 100% specificity [23]. However, the lack of standardization is a serious problem in the serological diagnosis of PCM, leading to discordant results that can even affect the patient’s treatment [24]. In addition, there is the possibility of cross-reaction with sera from patients with other mycoses, which may eventually reduce the specificity of the test [25].

Thus, the importance of developing new diagnostic methods for PCM is highlighted, even though it is considered a neglected tropical disease [26]. FTIR spectroscopy is a promising methodology, due to its ease of use, cost-effectiveness, and speed of analysis for a variety of clinical samples, with an emphasis on serum [27]. In the present study, we proposed two methods that use FTIR spectroscopy and serum for the diagnosis of PCM: one based on a univariate analysis, with 94% accuracy; and another based on a multivariate analysis, with 100% accuracy.

This was the first study to propose the use of FTIR and univariate analysis for the diagnosis of PCM, considering specific vibrational modes in the serum that differentiate these patients from healthy individuals and from patients with other systemic mycoses. The association of FTIR spectra with univariate analysis, to determine the vibrational modes capable of being used as diagnostic tools, has been little explored in the literature. Studies were found that used this type of analysis for the diagnosis of breast cancer in saliva, obtaining an accuracy that ranged from 77% to 83.5% [28]; for the diagnosis and monitoring of diabetes mellitus in nail clippings, obtaining an accuracy of 92% [29]; for monitoring diabetes mellitus during treatment with insulin, in saliva, obtaining an accuracy of 98.8% [30]; and for the diagnosis of chronic kidney disease (CKD) in saliva, obtaining an accuracy of 82% [31]. These studies demonstrate that univariate analyses of FTIR spectra are simple and fast methods that can achieve high levels of accuracy.

We confirmed the diagnostic potential of the wavenumber 1264 cm^−1^ from a ROC curve, which graphically demonstrated the rate of true positives (sensitivity) versus the rate of false positives (1-specificity) (Figure 3) [22]. For each absorbance value at this wavenumber, the sensitivity and specificity are shown graphically on the ROC curve, considering the objective of the analysis (in the case of the present study, differentiating PCM samples from samples from the control group). The goal is to find a value that maximizes the true positive rate while minimizing the false positive rate. Therefore, the objective is to find the value that best differentiates the samples and, consequently, maximizes accuracy (represented in the ROC curve by the area under the curve—AUC). The point that maximizes the accuracy is calculated using the Youden index [22], which determines, therefore, the ideal cutoff point to, in the case of the present study, classify the samples into each of the groups (PCM and control).

However, univariate analysis has its limitations, as it is subject to experimental variations and does not consider the dependencies between wavenumbers, which makes the developed model less robust [18]. In addition, despite having shown high sensitivity, greater than 97%, the specificity was lower, 76%. High sensitivity is an optimal feature for screening tests, while high specificity is an optimal feature for confirmatory testing [22]. Considering that the diagnosis of PCM is usually initiated by the identification of clinical symptoms, and that these symptoms can be confused with other diseases [1,7], specificity is also important. Thus, we also proposed a diagnostic method based on multivariate analysis, which considers multiple variables for the outcome of interest [32]. We used LDA, which is a powerful methodology, to perform class separation [18].

For LDA, the number of variables cannot be greater than the number of samples. Thus, it is often used together with some method of dimensionality reduction and variable selection, such as PCA [33]. Here, we started the analysis with univariate statistics and, based on this, selected a relevant spectral region for the performance of PCA. Thus, the PCA showed high variance (97% in PC1) and we achieved a complete separation of the groups. This allowed us, instead of using the PC1 and PC2 scores, to select from the PC1 loadings the most relevant wavenumbers to be used in the LDA. As an advantage, the variables were already selected and it is not always necessary to perform a PCA before applying the LDA.

The diagnosis of PCM based on FTIR and multivariate analysis methods has already been proposed in two other studies, which obtained 91.67% accuracy [16] and 100% accuracy [17]. The first [16] showed three main limitations in relation to the present study: (1) a small number of samples (only 20 samples from patients with PCM and 20 control subjects), (2) the model was only tested by cross-validation, without external validation, and (3) samples from patients with other systemic mycoses were not included. The second was developed by our research group, using OPLS-DA and considering the complete spectral region (4000–650 cm^−1^) [17]. Even having already obtained 100% accuracy in this previous model, in the present work we proposed a simpler model, with the use of LDA coupled to a variable selection method, which made it possible to replace the use of the total spectrum with only eight variables. This increases the robustness of the diagnostic model, maximizing the chance of a correct prediction of new external samples and making the model less sensitive to interference from environmental noise.

Thus, we identified some serum ATR/FTIR spectral biomarkers for PCM screening. Serum is a complex biofluid that perfuses organs throughout the body, bringing information about intra- and extracellular events [34]; therefore, it is useful for the diagnosis of various diseases. For PCM, the spectral biomarker in serum that showed the highest potential in univariate analysis was the 1264 cm^−1^ vibrational mode, with a significantly lower absorbance in PCM patients (Figure 3B and Figure 4B). This region of the spectrum is mainly associated with asymmetrical vibrations of PO4^−^, which is attributed to phosphate [25]. PCM patients may have reduced serum phosphate levels [1], which could explain the potential use of these vibrational modes for PCM diagnosis. The eight wavenumbers used in LDA, between 1500 and 1400 cm^−1^, mainly involve the carbon bonds present in lipids [35]. Different sphingolipids, such as cerebroside D, have already been selected as potential diagnostic biomarkers of PCM in serum through high-resolution mass spectrometry [36]. These sphingolipids participate in the antigenicity of *Paracoccidioides* spp., which could explain their potential as biomarkers [37]. However, fungal diseases are still poorly explored using FTIR spectroscopy, and the physiological explanation of these potential biomarkers still needs to be better explored.

## 5. Conclusions

Here, simple and low-cost diagnostic methods for PCM, based on FTIR, were proposed. Its main advantages over existing methods are its speed of analysis, the minimal generation of waste, and the use of serum, a minimally invasive sample that is easily collected from most patients. LDA modeling was highlighted, which achieved 100% accuracy, showing a better performance than routine PCM diagnostic methods. In addition, it was possible to identify spectral biomarkers that differentiate the serum from patients with PCM from that from patients with other systemic mycoses, which can be confounded by clinical signs and serology. In this way, we have proved the potential of the methodology proposed here to be applied to the diagnosis of PCM.

## Figures and Tables

**Table 1:** Area under the curve (AUC) obtained in the individual ROC curves developed with all wavenumbers which had *p* < 0.05 in the *t*-test, comparing the spectra of the 92 serum samples from the control group and the 132 samples from the paracoccidioidomycosis (PCM) group. The control group is composed of serum from 50 healthy blood donors, 24 patients with aspergillosis, 10 patients with cryptococcosis, and 8 patients with histoplasmosis. Data are represented in spectrum ranges and AUC ranges for better data visualization.

Interval (cm^−1^)	AUC–ROC Curve (Range)
3813–2965	0.11512–0.47785
2932–2908	0.62459–0.65514
2869–2843	0.65201–0.68248
1752–1742	0.60713–0.62731
1726–1686	0.35787–0.48271
1664–1638	0.61421–0.73814
1630–1594	0.21665–0.50091
1571–1556	0.31308–0.48295
1551–1095	0.57617–0.94219
1041–1032	0.39295–0.40110
1027–846	0.29076–0.37039
830–715	0.32633–0.37788

Abbreviations: AUC—area under the curve; ROC—receiver operating characteristic.

## Data Availability

The datasets presented in this article are not readily available because the data are part of an ongoing study. Requests to access the datasets should be directed to the corresponding author.
